# Spontaneous subcapsular hepatic hematoma with hepatic rupture and hemorrhage in the postpartum period: a case report and literature review

**DOI:** 10.3389/fmed.2026.1823131

**Published:** 2026-05-04

**Authors:** Penghui Liu, Na Li, Jiwu Guo, Jizhen Wang, Ziyuan Mou, Jie Mao

**Affiliations:** Department of General Surgery, Lanzhou University Second Hospital, Lanzhou, China

**Keywords:** hepatic hemorrhage, hepatic rupture, postpartum, subcapsular hepatic hematoma, transcatheter arterial embolization

## Abstract

Subcapsular hepatic hematoma with hepatic rupture in the postpartum period is a rare but potentially fatal condition, most commonly associated with hypertensive disorders of pregnancy and HELLP syndrome. In women without typical risk factors, non-specific symptoms may delay diagnosis. We report a previously healthy term primiparous woman who developed sudden, persistent right upper abdominal pain with nausea 3 h after vaginal delivery. On admission, she had right upper abdominal tenderness, tachycardia, and relative hypotension. Laboratory tests showed anemia, markedly elevated transaminases, and mild coagulation abnormalities. Contrast-enhanced CT demonstrated a large right-lobe subcapsular hepatic hematoma (14.8 × 12.4 cm) with hepatic parenchymal laceration and contrast extravasation, indicating active bleeding, together with perihepatic and abdominopelvic fluid. Emergency angiography confirmed multifocal bleeding from hepatic arterial branches with a hepatic artery–portal vein shunt, and superselective transcatheter arterial embolization achieved hemostasis. Because CT and pelvic angiography raised concern for a possible concurrent occult uterine arterial bleeding source, adjunct bilateral uterine artery embolization was performed in the same session. Recovery was uneventful, with progressive clinical and laboratory improvement. At 5-month follow-up, imaging showed marked hematoma resolution without rebleeding or related complications. This case highlights that persistent postpartum upper abdominal pain warrants early imaging evaluation even in the absence of typical HDP/HELLP-related risk factors. In selected patients with imaging evidence of active bleeding who remain suitable for urgent angiographic intervention, transcatheter arterial embolization may provide an effective minimally invasive hemostatic option.

## Introduction

Postpartum subcapsular hepatic hematoma with concomitant hepatic rupture and hemorrhage is a rare but life-threatening obstetric complication (1). Delayed diagnosis and treatment may lead to rapid progression to hemorrhagic shock and even death (2). Previous reports suggest that this condition is most commonly associated with hypertensive disorders of pregnancy, HELLP syndrome, coagulation abnormalities, or underlying liver disease (3). However, in women without typical risk factors, the initial presentation is often limited to persistent epigastric or right upper abdominal pain, which can be misattributed to gastrointestinal dysfunction, gallbladder disease, or normal postpartum uterine involution, thereby resulting in misdiagnosis and delayed recognition (4).

Most reported cases of subcapsular hepatic hematoma and hepatic rupture in the postpartum period have been described in association with hypertensive disorders of pregnancy, particularly preeclampsia and HELLP syndrome (3, 5, 6). In contrast, postpartum cases occurring in previously normotensive women without clear clinical or laboratory evidence of HELLP appear to be distinctly uncommon (7). In the present case, a previously healthy woman developed persistent right upper abdominal pain shortly after delivery (8). Contrast-enhanced CT revealed a subcapsular hematoma in the right hepatic lobe with parenchymal laceration and imaging evidence of active bleeding (9). Following multidisciplinary evaluation, transcatheter hepatic arteriography was performed to identify the culprit vessels, and selective embolization achieved successful hemostasis with a favorable clinical course on follow-up (10). Herein, we report this case not only to improve early recognition of postpartum subcapsular hepatic hematoma and hepatic rupture with hemorrhage, but also to discuss individualized management according to hemodynamic status, imaging evidence of active bleeding, and the possibility of concomitant extrahepatic bleeding sources (6, 11).

## Case presentation

### General information and medical history

A 24-year-old primiparous woman at 39 + 4 weeks’ gestation delivered a live infant via vaginal delivery at an outside hospital; the delivery was uneventful. She had been previously healthy, with no history of chronic liver disease, hypertension, diabetes mellitus, or coagulation disorders, and no history of long-term medication use or abdominal trauma. She attended regular prenatal visits. Her blood pressure remained normal throughout pregnancy, urinalysis was negative for proteinuria, and there were no documented abnormalities in liver function or thrombocytopenia during pregnancy.

Approximately 3 h postpartum, she developed sudden-onset, persistent right upper abdominal pain described as a dull ache radiating to the back, accompanied by mild nausea. She denied vomiting, melena, gross hematuria, jaundice, or altered mental status. Postpartum lochia was bloody in appearance but within the expected volume and characteristics in the postpartum period, without ongoing heavy vaginal bleeding. An emergent abdominal ultrasound performed at the outside hospital suggested possible hepatic bleeding, and she was therefore transferred emergently to our hospital by ambulance for further evaluation and management. Before and during transfer, supportive treatment including intravenous fluid infusion was provided. No blood transfusion, endotracheal intubation, or cardiopulmonary resuscitation was performed before transfer. Although her blood pressure and overall vital signs had been suboptimal at the referring hospital, no obvious clinical deterioration occurred during transport.

### Physical examination

On admission, the patient was in fair general condition. Vital signs were as follows: temperature 39.0°C, pulse 120 beats/min, respiratory rate 24 breaths/min, and blood pressure 95/75 mmHg. No scleral icterus was noted. The abdomen was flat with tenderness in the right upper abdomen and mild rebound tenderness, without obvious muscular guarding. Percussion tenderness over the hepatic area was negative. Bowel sounds were decreased (approximately 2/min).

Obstetric examination revealed a uterine fundal height consistent with a term primiparous uterus; uterine contraction was adequate. A small amount of bloody lochia was present without foul odor.

### Laboratory examination

At admission/preoperatively: Hb 93 g/L and PLT 113 × 10^9/L; ALT 2248 U/L, AST 3582 U/L, TBiL 54.5 μmol/L, and ALB 32.4 g/L; PT 16.4 s, INR 1.46, APTT 30.1 s, Fib 2.49 g/L, and D-dimer 84.54 mg/L.

At 24 h postoperatively: Hb 79 g/L and PLT 153 × 10^9/L; ALT 901 U/L, AST 604 U/L, TBiL 47.5 μmol/L, and ALB 30.0 g/L; PT 13.3 s, INR 1.18, APTT 28.7 s, Fib 2.88 g/L, and D-dimer 46.08 mg/L.

Before discharge: Hb 97 g/L and PLT 253 × 10^9/L; ALT 58 U/L, AST 76 U/L, TBiL 22.8 μmol/L, and ALB 32.1 g/L; PT 11.5 s, INR 1.06, APTT 29.6 s, Fib 2.98 g/L, and D-dimer 32.59 mg/L.

Overall, these findings did not support hemolysis, thrombocytopenia, or progressive liver failure. There were no typical laboratory features of HELLP syndrome, acute fatty liver of pregnancy, or disseminated intravascular coagulation, and no clear evidence of infection.

### Imaging examination

**FIGURE 1:**
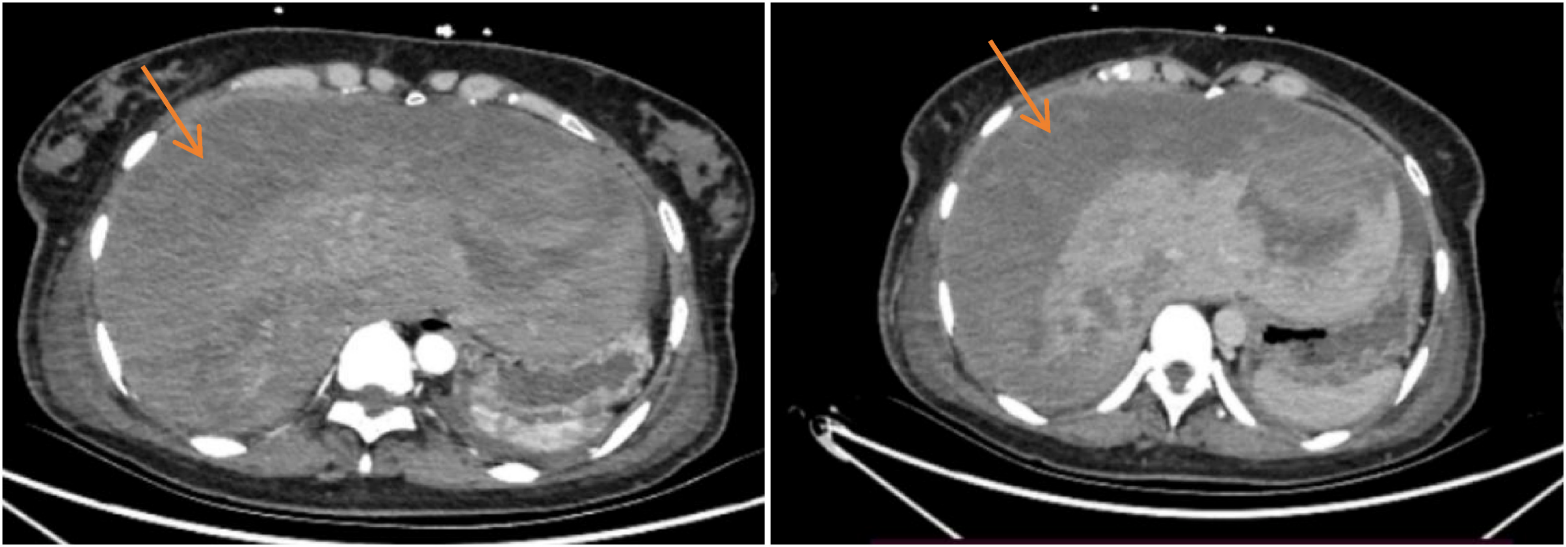
Admission contrast-enhanced abdominal CT. Arterial-phase contrast-enhanced abdominal CT obtained on admission demonstrated a large mixed-attenuation subcapsular hematoma in the right hepatic lobe with capsular bulging (arrow). A focal hepatic parenchymal laceration and punctate/patchy contrast extravasation were also identified, indicating active hepatic bleeding. Associated perihepatic fluid and abdominopelvic fluid were present, supporting the diagnosis of subcapsular hepatic hematoma with hepatic rupture and hemorrhage.

**FIGURE 2:**
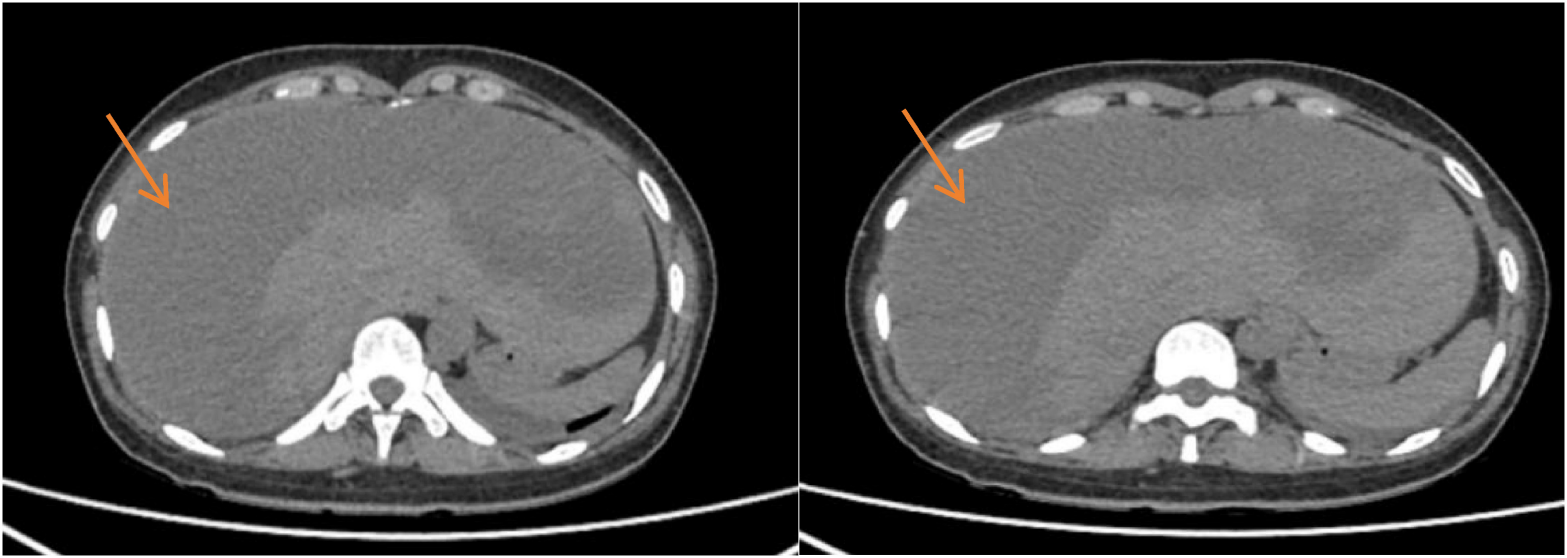
Follow-up abdominal CT after embolization (postoperative day 7/pre-discharge). Contrast-enhanced CT performed 7 days after transcatheter arterial embolization showed a slight reduction in the size of the right-lobe subcapsular hematoma (arrow) with decreased attenuation compared with admission. No new contrast extravasation, expanding hemoperitoneum, or progressive intraperitoneal fluid collection was observed, indicating successful hemostatic control.

(1) Abdominal ultrasound at the outside hospital: Abnormal hepatic parenchymal echogenicity with a suspected subcapsular hematoma; hepatic bleeding was considered.
(2) Contrast-enhanced abdominal CT on admission (preoperatively): A subcapsular hematoma in the right hepatic lobe was identified (maximum diameter approximately 14.8 × 12.4 cm) with associated hepatic parenchymal laceration. Arterial-phase punctate/patchy contrast extravasation was noted, indicating active bleeding. Perihepatic hematoma/fluid as well as abdominopelvic fluid were present. The postpartum uterus was enlarged with a small amount of intrauterine fluid/gas, which was considered consistent with postpartum changes. An incidentally detected abnormally enhancing focus in the left adnexal region was also noted; correlation with obstetric examination and follow-up evaluation was recommended (Figure 1).
(3) Early post-embolization reassessment imaging: On postoperative day 1, bedside ultrasonography showed a slight reduction in the extent of the right-lobe subcapsular hematoma, with decreased and more homogeneous internal echogenicity; no increase in newly developed perilesional anechoic fluid was observed. Color Doppler imaging showed no definite flow signal suggestive of ongoing active bleeding, indicating effective hemostatic control. On postoperative day 7 (pre-discharge), follow-up abdominal CT demonstrated a slight decrease in hematoma volume and lower attenuation compared with prior imaging, without new contrast extravasation. No new intraperitoneal free hemorrhage or progressively increasing fluid was observed, confirming effective control of acute bleeding and successful embolization. Post–uterine artery embolization changes and a small amount of pelvic fluid were also noted (Figure 2).

## Diagnosis and differential diagnosis

1. Clinical diagnosis

- Postpartum subcapsular hepatic hematoma.
- Hepatic rupture with active hemorrhage.
- Angiographic contrast extravasation from the uterine arteries, raising concern for a possible occult pelvic arterial bleeding source; adjunct bilateral uterine artery embolization was performed after multidisciplinary assessment.
- Anemia (Hb 93 g/L).

2. Key points of differential diagnosis

(1) HELLP syndrome/hypertensive disorders of pregnancy–related hepatic injury: Typically characterized by hypertension, proteinuria, hemolysis (e.g., elevated LDH and indirect bilirubin), thrombocytopenia, and markedly elevated liver enzymes. In this patient, blood pressure and proteinuria were normal during pregnancy, and laboratory tests showed no clear evidence of hemolysis or thrombocytopenia; liver function and coagulation parameters did not show progressive deterioration. Therefore, this diagnosis was considered unlikely.
(2) Acute fatty liver of pregnancy (AFLP): Often presents with progressive jaundice, hypoglycemia, marked coagulopathy, and multi-organ dysfunction. The patient had no obvious jaundice and only mild coagulation abnormalities, without typical features, making AFLP less likely.
(3) Severe disseminated intravascular coagulation (DIC): Usually manifests as progressive coagulopathy (marked prolongation of PT/APTT and a significant decrease in fibrinogen) with a bleeding tendency. In this case, coagulation abnormalities were mild and non-progressive, and typical laboratory features of DIC were absent.
(4) Traumatic/iatrogenic hepatic injury: The patient denied abdominal trauma or prior invasive hepatic procedures, and imaging findings were not consistent with a traumatic mechanism; thus, this etiology was not supported.
(5) Other causes of postpartum upper abdominal pain: Including acute cholecystitis, pancreatitis, and gastrointestinal perforation. These were excluded or considered unlikely based on contrast-enhanced CT findings and the corresponding clinical presentation.

## Treatment course

**FIGURE 3:**
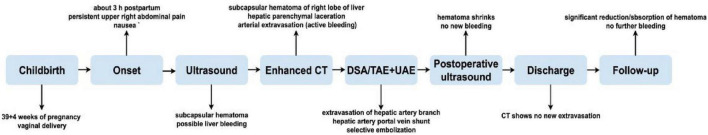
Timeline of the clinical course and key diagnostic and therapeutic milestones. The timeline summarizes symptom onset shortly after vaginal delivery, transfer to our hospital, admission imaging findings, emergency angiography, superselective hepatic arterial embolization, adjunct bilateral uterine artery embolization, postoperative reassessment, discharge, and follow-up. DSA, digital subtraction angiography; TAE, transcatheter arterial embolization; UAE, uterine artery embolization.

After multidisciplinary consultation involving obstetrics, hepatobiliary surgery, interventional radiology, and anesthesiology, the patient was considered to have borderline hemodynamic compromise rather than unequivocal stability, as reflected by tachycardia and relative hypotension on admission. Nevertheless, she had not progressed to circulatory collapse, and contrast-enhanced abdominal CT had demonstrated a large subcapsular hematoma in the right hepatic lobe with parenchymal laceration and imaging evidence of active bleeding, indicating a risk of ongoing hemorrhage and further hematoma rupture. Urgent transcatheter angiography with selective embolization was therefore planned for hemostasis. The clinical course and key diagnostic/therapeutic milestones are summarized in Figure 3.

### Transcatheter arterial embolization (TAE)

Under local anesthesia, the right common femoral artery was accessed using the Seldinger technique, and a 5-F arterial sheath was placed. A 5-F RH catheter was advanced for celiac trunk and hepatic arteriography. Angiography demonstrated multiple small, nodular foci of contrast extravasation in both hepatic lobes (predominantly from right-lobe branches), accompanied by a hepatic artery–portal vein shunt. The shunt was presumed to be secondary to injury of the intrahepatic vascular bed. To preserve perfusion to uninvolved liver parenchyma as much as possible, a microcatheter was used to superselectively catheterize the culprit bleeding branches, and selective embolization was performed in a staged, low-flow manner using a temperature-sensitive liquid embolic agent combined with gelatin sponge particles. Repeat angiography showed marked slowing or interruption of flow in the target branches with disappearance of contrast extravasation, while perfusion of the main hepatic artery was preserved, indicating effective hemostasis.

### Additional pelvic vascular assessment and uterine artery embolization (UAE)

Given the preprocedural CT finding of abdominopelvic fluid, additional pelvic angiographic evaluation was performed after hepatic embolization to assess for a potential concurrent pelvic arterial bleeding source. The catheter was exchanged for a 4-F Cobra catheter, and bilateral internal iliac arteriography followed by bilateral uterine arteriography was performed. Angiography demonstrated enlarged and tortuous uterine arteries with abnormal hypervascularity and contrast extravasation from both uterine arteries, more pronounced on the left. Although the patient did not have persistent heavy vaginal bleeding clinically and the lochia remained within the expected postpartum range, these angiographic findings, together with the abdominopelvic fluid seen on CT, raised concern for a possible occult uterine arterial bleeding source. After multidisciplinary discussion, adjunct bilateral UAE was therefore performed via superselective microcatheterization using 560–710 μm gelatin sponge particles. Post-embolization angiography confirmed cessation of distal uterine arterial opacification. We acknowledge that, in the absence of overt postpartum hemorrhage, the clinical significance of these angiographic findings cannot be established with absolute certainty; however, they were considered sufficiently suspicious to justify prophylactic/therapeutic treatment in the setting of postpartum acute abdomen and possible multifocal bleeding.

At the end of the procedure, the catheter and sheath were removed, hemostasis was achieved with local compression/closure and pressure dressing, and immobilization of the right lower extremity was advised. Postoperatively, the patient was closely monitored and received supportive care including fluid resuscitation, analgesia, hepatoprotective therapy, and correction of coagulopathy. Packed red blood cells and/or plasma were transfused according to hemoglobin and coagulation results. Abdominal pain, peritoneal signs, and lochia were reassessed continuously, and complete blood count, liver function tests, and coagulation parameters were monitored dynamically.

## Outcome and follow-up

After the procedure, the patient was managed in the general ward with close monitoring rather than in the intensive care unit. Within 24 h after the procedure, her vital signs remained stable. The volume and characteristics of lochia were within the expected range in the postpartum period, and there was no persistent heavy vaginal bleeding. Laboratory tests at 24 h postoperatively showed: Hb 79 g/L and PLT 153 × 10^9/L; liver function indices: ALT 901 U/L, AST 604 U/L, TBiL 47.5 μmol/L, and ALB 30.0 g/L; coagulation profile: PT 13.3 s, INR 1.18, APTT 28.7 s, Fib 2.88 g/L, and D-dimer 46.08 mg/L. Bedside ultrasonography/CT reassessment demonstrated a decrease in the size of the subcapsular hematoma in the right hepatic lobe, with reduced internal echogenicity/attenuation compared with prior imaging. No new signs of active bleeding or intraperitoneal free hemorrhage were identified, confirming effective hemostasis after embolization.

During the 7-day hospitalization, the overall course was smooth. The patient developed fever, but no evidence of severe infection, recurrent bleeding, hemodynamic deterioration, worsening jaundice, peritonitis, or other major complications was observed. Complete blood count, liver function tests, and coagulation parameters were monitored dynamically and gradually improved. She was discharged in stable condition on postoperative day 7. At discharge, Hb was 97 g/L, liver function and coagulation parameters had improved markedly, and abdominal pain was significantly relieved.

**FIGURE 4:**
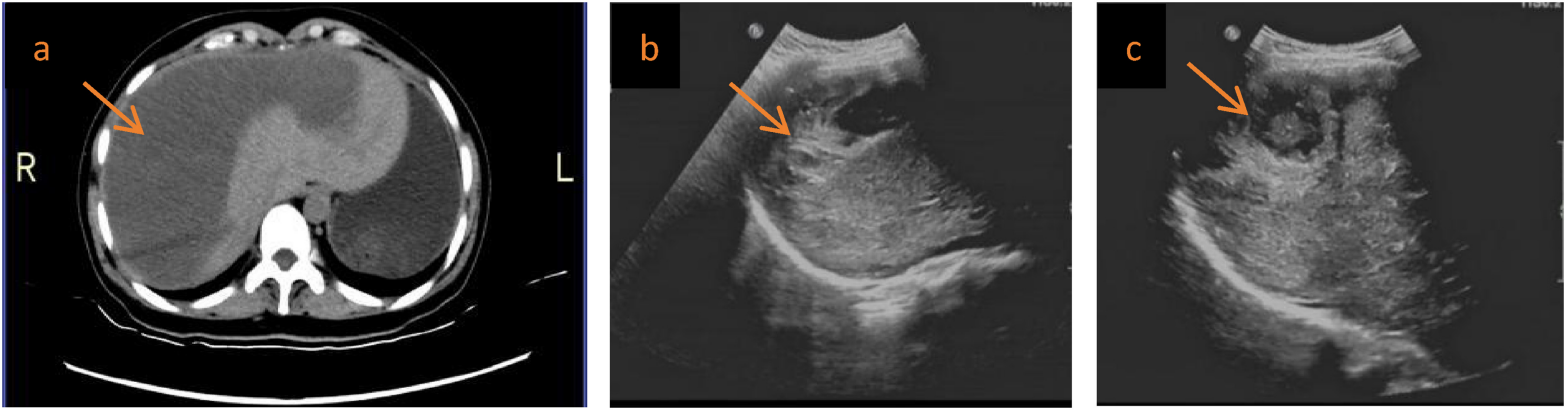
Two-month follow-up imaging after embolization. **(a)** Follow-up abdominal CT at 2 months showed marked reduction in the size of the right-lobe subcapsular hematoma compared with admission (arrow), with lower attenuation and less capsular bulging, without imaging evidence of recurrent active bleeding. **(b,c)** Hepatic ultrasonography at the same time point demonstrated residual strip-like and arcuate mildly hyperechoic subcapsular areas in the right hepatic lobe (arrow), consistent with organizing and resorbing hematoma. The surrounding hepatic parenchyma appeared homogeneous, and no obvious intraperitoneal free fluid was detected.

**FIGURE 5:**
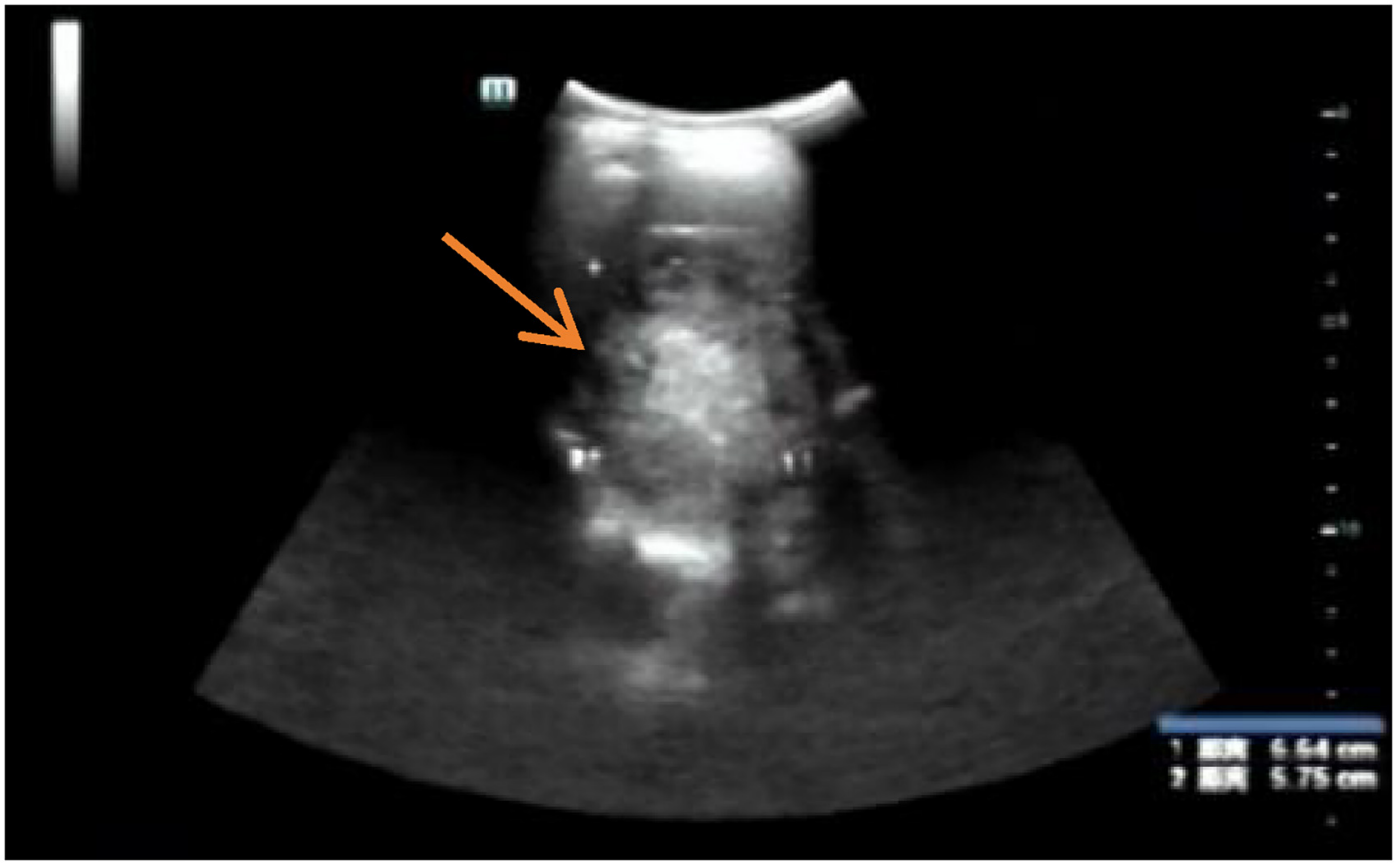
Five-month follow-up ultrasound. Ultrasonography obtained 5 months after embolization showed a residual mixed-echogenic lesion in hepatic segment VII without detectable internal Doppler flow, compatible with resolving post-hemorrhagic and post-embolization change. No sonographic evidence of recurrent hemorrhage or new perihepatic fluid collection was observed.

After discharge, regular follow-up was arranged (Figure 4). The patient was advised to avoid strenuous activity and to undergo periodic reassessment of complete blood count, liver function, and imaging. At the 5-month follow-up, she remained in good general condition with no recurrent abdominal pain or bleeding-related symptoms. Follow-up imaging showed marked reduction/resorption of the subcapsular hepatic hematoma, with gradual resolution of the hepatic laceration area, and no evidence of rebleeding or related complications; follow-up is ongoing (Figure 5 and Table 1).

**TABLE 1:** Key laboratory and imaging parameters at predefined time points.

Indicator (unit)	Reference range	Admission/Preoperative	24-h postoperative	Discharge	Follow up 2 months	Follow up 5 months
BP (mmHg)	Normal < 120/ < 80	95/75	110/84	113/78	112/75	107/74
Hb (g/L)	110–150	93	79	97	110	133
PLT (× 10^9/L)	100–300	113	153	253	265	259
ALT (U/L)	7–40	2,248	901	58	35	29
AST (U/L)	13–35	3,582	604	76	27	21
TBiL (μmol/L)	≤ 21	54.5	47.5	22.8	17.9	13.3
ALB (g/L)	40.0–55.0	32.4	30.0	32.1	41.1	47.0
PT (s)	9.4–12.5	16.4	13.3	11.5	10.9	11.0
INR	0.85–1.14	1.46	1.18	1.06	1.01	1.01
APTT (s)	25.4–38.4	30.1	28.7	29.6	30.6	30.7
Fib (g/L)	2.00–5.00	2.49	2.88	2.98	2.99	3.00
D-dimer (mg/L)	< 0.50	84.54	46.08	32.59	3.06	< 0.50

BP, blood pressure; Hb, hemoglobin; PLT, platelet count; ALT, alanine aminotransferase; AST, aspartate aminotransferase; TBiL, total bilirubin; ALB, albumin; PT, prothrombin time; INR, international normalized ratio; APTT, activated partial thromboplastin time; Fib, fibrinogen. D-dimer values are reported as “ < 0.50” when below the assay detection limit.

## Discussion

Subcapsular hepatic hematoma with concomitant hepatic rupture and hemorrhage in the postpartum period is rare in clinical practice. In patients presenting with persistent epigastric/right upper abdominal pain, progressive anemia, and imaging evidence of a subcapsular hepatic hematoma or hepatic parenchymal laceration, clinicians should maintain a high index of suspicion and promptly assess the risk of ongoing bleeding (5).

Most published cases of subcapsular hepatic hematoma and hepatic rupture in the postpartum period have been reported in association with hypertensive disorders of pregnancy, particularly preeclampsia and HELLP syndrome (3, 5, 6). In the present case, however, the patient lacked typical clinical and laboratory features of hypertensive disorders of pregnancy or HELLP syndrome during pregnancy and postpartum, placing this case within the less common spectrum of spontaneous postpartum subcapsular hepatic hematoma (7). The pathophysiology in such non-HDP/non-HELLP postpartum cases remains incompletely understood and is likely multifactorial (3, 6). During pregnancy, increases in circulating blood volume and cardiac output, together with augmented hepatic perfusion, may render the hepatic microvasculature more vulnerable to injury (1, 3). In addition, labor and the immediate postpartum period are associated with abrupt fluctuations in venous return and intra-abdominal pressure, which may increase mechanical stress on subcapsular vessels and contribute to hematoma formation or rupture (2, 3). Pregnancy-related hormonal and hemostatic changes may further affect vascular wall integrity and the balance between coagulation and fibrinolysis, although these mechanisms remain hypothetical in women without HDP/HELLP (1, 3). Although these hypotheses require further validation in larger case series and mechanistic studies, they highlight that persistent or progressively worsening upper abdominal pain after delivery should not be routinely attributed to common gastrointestinal or biliary disorders, even in otherwise healthy postpartum women (12).

An additional practical lesson from this case lies in the differential diagnosis of postpartum upper abdominal pain. In the postpartum setting, clinical presentations may overlap considerably among HELLP syndrome–related hepatic injury, acute fatty liver of pregnancy, disseminated intravascular coagulation, traumatic or iatrogenic hepatic injury, and common non-obstetric conditions such as acute cholecystitis, pancreatitis, or gastrointestinal perforation. In our patient, the absence of hypertension, proteinuria, hemolysis, progressive thrombocytopenia, severe hypoglycemia, or progressive coagulopathy, together with contrast-enhanced CT findings of subcapsular hematoma, hepatic parenchymal laceration, and active contrast extravasation, helped narrow the differential diagnosis and supported prompt recognition of postpartum subcapsular hepatic hematoma with rupture and hemorrhage. This diagnostic reasoning may be particularly important in atypical postpartum cases without classic HDP/HELLP features.

Clinically, the initial symptom of postpartum subcapsular hepatic hematoma is most often upper abdominal or right upper abdominal pain, sometimes accompanied by nausea and vomiting; severe cases may present with hemorrhagic shock (4). Because postpartum abdominal pain is frequently attributed to gastrointestinal dysfunction, gallbladder disease, or non-specific postpartum discomfort, misdiagnosis and delayed recognition may occur (12). Therefore, for postpartum women with unexplained persistent or progressively worsening upper abdominal pain—particularly when accompanied by anemia, right upper abdominal tenderness, or signs of peritoneal irritation—early imaging evaluation is warranted (9). Ultrasonography is useful as a bedside screening tool, but has limited capability in delineating the extent of laceration and detecting active bleeding. In contrast, contrast-enhanced CT can rapidly identify subcapsular hematoma, hepatic parenchymal laceration, and arterial-phase contrast extravasation as evidence of active hemorrhage, and can also assess hemoperitoneum or ascites, thereby providing critical information for treatment decision-making (9).

Management should be individualized according to the hemodynamic trajectory, hematoma extent, rupture severity, and the presence of imaging evidence of active bleeding (3, 6). In carefully selected patients with relatively small or contained hematomas, no imaging evidence of active hemorrhage, and sustained hemodynamic stability under close monitoring, conservative management may be appropriate (6, 7). However, when imaging demonstrates active bleeding or there is an ongoing risk of blood loss, timely intervention is required (6, 13). Traditional surgery allows direct hemostasis and management of hepatic rupture, but it is invasive and associated with substantial perioperative risk (6, 14). With advances in interventional radiology, transcatheter arterial embolization enables precise localization of culprit vessels and superselective embolization under imaging guidance, achieving rapid hemostasis while preserving perfusion to uninvolved liver parenchyma whenever possible (10). This approach is minimally invasive, effective, and repeatable, and is particularly suitable for patients who remain sufficiently compensated to undergo urgent angiographic intervention despite imaging evidence of active bleeding (6, 10). In our case, selective embolization of the culprit hepatic arterial branches eliminated contrast extravasation on follow-up imaging, indicating effective hemostasis (10). No rebleeding or clinically significant hepatic dysfunction was observed during follow-up, supporting the feasibility and safety of this strategy under comprehensive assessment and multidisciplinary collaboration (8, 10).

A brief comparison with the published literature provides additional context for the present case. First, most reported cases of pregnancy- or postpartum-associated subcapsular hepatic hematoma and hepatic rupture have occurred in association with preeclampsia and/or HELLP syndrome, whereas postpartum presentations in previously normotensive women without clear HELLP features appear to be distinctly uncommon (3, 5, 6). Second, reported management strategies remain heterogeneous, ranging from conservative monitoring to surgery and endovascular embolization, with treatment choice primarily driven by hemodynamic status, rupture severity, and imaging evidence of ongoing hemorrhage (6, 15). Third, although the experience with hepatic arterial embolization remains limited compared with surgery or conservative management, recent reports continue to support TAE as a useful less invasive option in selected patients with treatable arterial bleeding (10, 16). In contrast, pelvic embolization is already well established in obstetric hemorrhage management, which provides indirect support for adjunct UAE when angiography suggests a second suspected pelvic arterial source (17). Representative previously reported cases and their management strategies are summarized in Supplementary Table 1.

A particularly debatable aspect of this case is the decision to perform adjunct uterine artery embolization despite the absence of persistent heavy vaginal bleeding. In the present patient, preprocedural imaging suggested intraperitoneal or abdominopelvic fluid (18). After hepatic embolization, additional angiographic assessment of the bilateral internal iliac and uterine arteries revealed enlarged, tortuous uterine arteries with contrast extravasation, more pronounced on the left, raising concern for a possible concurrent occult uterine arterial bleeding source (17). Adjunct bilateral uterine artery embolization was therefore performed after multidisciplinary assessment (17). Although the patient did not exhibit persistent heavy vaginal bleeding clinically and lochia remained within the expected postpartum range, these angiographic findings, together with the abdominopelvic fluid seen on CT, supported a systematic evaluation for possible multifocal bleeding sources in a postpartum patient presenting with an acute abdomen (5, 17). Because overt postpartum hemorrhage was not clinically evident, the true clinical significance of the uterine angiographic abnormality cannot be established with absolute certainty; therefore, the adjunct UAE should be interpreted as a cautious same-session treatment of a second suspected arterial source rather than definitive proof of clinically significant uterine bleeding (17). This report was prepared in accordance with the CARE guidelines for case reports (19).

## Conclusion

In summary, this case highlights that postpartum subcapsular hepatic hematoma with hepatic rupture and hemorrhage may occur even in previously healthy women without typical HDP/HELLP-related risk factors. Persistent postpartum upper abdominal pain should prompt early imaging evaluation, especially when accompanied by anemia, tenderness, or relative hemodynamic compromise. In selected patients with imaging evidence of active bleeding who remain suitable for urgent angiographic intervention, superselective transcatheter hepatic arterial embolization may provide an effective minimally invasive hemostatic option. This case also underscores the importance of individualized multidisciplinary assessment, particularly when concomitant extrahepatic bleeding sources are suspected but not clinically overt.

## Data Availability

The original contributions presented in this study are included in the article/Supplementary material, further inquiries can be directed to the corresponding authors.
