# Supplementary material for: Spontaneous subcapsular hepatic hematoma with hepatic rupture and hemorrhage in the postpartum period: a case report and literature review
Source: Front Med (Lausanne). 2026 May 4;13:1823131. doi: 10.3389/fmed.2026.1823131 (PMC13180929; doi:10.3389/fmed.2026.1823131)
Supplement: Supplementary file 1 [file Table_1.docx]

**Supplementary Table S1. Representative previously reported cases of pregnancy- or postpartum-associated subcapsular hepatic hematoma/hepatic rupture, management strategies, and outcomes, compared with the present case**

| Study | Clinical setting / background | Rupture or active bleeding | Main management | Maternal outcome | Relevance to the present case |
| --- | --- | --- | --- | --- | --- |
| Marinaș et al., 2018 (current Ref. [7]) | Postpartum spontaneous subcapsular hepatic hematoma without associated maternal comorbidity; diagnosed after delivery | No overt rupture emphasized in the report | Conservative management | Recovered | Supports that postpartum spontaneous cases without clear HDP/HELLP do occur, but are uncommon and may be managed nonoperatively in selected stable patients |
| Anyfantakis et al., 2014 (current Ref. [12]) | Postpartum case related to preeclampsia; severe epigastric pain a few hours after emergency cesarean section | Subcapsular right-lobe hematoma; successfully managed without surgery | Conservative management | Recovered | Illustrates delayed postpartum recognition and successful conservative treatment in a preeclampsia-associated case |
| Luhning et al., 2021 (current Ref. [8]) | Severe preeclampsia/HELLP; persistent postpartum right-sided pain; CT showed a 16 cm subcapsular hepatic hematoma | Large unruptured hematoma | Initial conservative management; later video-assisted thoracoscopic surgery for infected/loculated pleural effusion | Discharged on postpartum day 21 | Shows that even unruptured hematomas can have a complicated postpartum course and require close follow-up despite initial conservative treatment |
| Nam et al., 2021 (current Ref. [10]) | HELLP syndrome at 28 weeks; hypotension, anemia, fetal death; rupture identified after cesarean section | Yes; spontaneous hepatic rupture with active bleeding | Hepatic packing plus transcatheter arterial embolization (TAE) | Uneventful postprocedural course; discharged 14 days after admission | Directly supports TAE as a less invasive hemostatic option in selected patients with treatable arterial bleeding |
| Chen et al., 2024 (current Ref. [15]) | Preeclampsia without HELLP; large hepatic subcapsular hematoma during pregnancy | Yes; ruptured during emergency cesarean section with major hemoperitoneum | Surgical hemostasis with packing | Complete recovery; hematoma reduction at 1-month follow-up | Particularly relevant for framing non-HELLP presentations and emphasizing that rupture can still occur outside classic HELLP syndrome |
| Zhou et al., 2024 (current Ref. [16]) | Postpartum HELLP syndrome after cesarean section; rapid blood-pressure drop and active liver-surface bleeding at laparotomy | Yes; spontaneous liver rupture/ongoing hemorrhage | Emergency TAE (with surgical exploration/laparotomy context) | Discharged 37 days after admission | Provides recent support for TAE in postpartum HELLP-associated rupture, reinforcing the role of endovascular hemostasis in selected unstable or borderline patients |
| Present case | Previously healthy postpartum woman without HDP/HELLP; symptoms began 3 hours after vaginal delivery | Yes; CT showed parenchymal laceration and contrast extravasation | Superselective hepatic TAE plus adjunct bilateral UAE after pelvic angiography suggested a second suspected occult bleeding source | Uneventful recovery; marked hematoma resolution without rebleeding at 5 months | Highlights the uncommon non-HDP/non-HELLP postpartum setting and the additional decision-making challenge posed by a possible concurrent pelvic arterial bleeding source |

Abbreviations: HDP, hypertensive disorders of pregnancy; HELLP, hemolysis, elevated liver enzymes, and low platelets; TAE, transcatheter arterial embolization; UAE, uterine artery embolization.
